# Inflammation and Pharmacological Treatment in Diabetic Retinopathy

**DOI:** 10.1155/2013/213130

**Published:** 2013-10-28

**Authors:** Snježana Kaštelan, Martina Tomić, Antonela Gverović Antunica, Jasminka Salopek Rabatić, Spomenka Ljubić

**Affiliations:** ^1^Department of Ophthalmology, Clinical Hospital Dubrava, Avenija Gojka Šuška 6, 10000 Zagreb, Croatia; ^2^Department of Ophthalmology, University Clinic Vuk Vrhovac, Clinical Hospital, Merkur, Zajčeva 19, 10000 Zagreb, Croatia; ^3^Department of Ophthalmology, General Hospital Dubrovnik, Dr. Roka Mišetića 2, 20000 Dubrovnik, Croatia; ^4^Department of Endocrinology and Metabolic Diseases, University Clinic Vuk, Vrhovac, Clinical Hospital Merkur, Zajčeva 19, 10000 Zagreb, Croatia

## Abstract

Diabetic retinopathy (DR), the most common microvascular complication of diabetes mellitus, is estimated to be the leading cause of new blindness in the working population of developed countries. Primary interventions such as intensive glycemic control, strict blood pressure regulation, and lipid-modifying therapy as well as local ocular treatment (laser photocoagulation and pars plana vitrectomy) can significantly reduce the risk of retinopathy occurrence and progression. Considering the limitations of current DR treatments development of new therapeutic strategies, it becomes necessary to focus on pharmacological treatment. Currently, there is increasing evidence that inflammatory processes have a considerable role in the pathogenesis of DR with multiple studies showing an association of various systemic as well as local (vitreous and aqueous fluid) inflammatory factors and the progression of DR. Since inflammation is identified as a relevant mechanism, significant effort has been directed to the development of new concepts for the prevention and treatment of DR acting on the inflammatory processes and the use of pharmacological agents with anti-inflammatory effect. Inhibiting the inflammatory pathway could be an appealing treatment option for DR in future practices, and as further prospective randomized clinical trials accumulate data, the role and guidelines of anti-inflammatory pharmacologic treatments will become clearer.

## 1. Introduction

Diabetes mellitus is the most frequent endocrine disease in developed countries estimated to have affected 366 million people worldwide and is expected to nearly double by 2030 owing to an increase in obesity, life span extension, and better detection of the disease. This global increase has a significant impact on the prevalence of diabetic complications among which diabetic retinopathy (DR) takes an important place [1, 2]. DR is a leading cause of acquired blindness in working-age adults and has been estimated to represent 12% of blindness in developed countries [3, 4]. The prevalence of retinopathy increases with the duration of diabetes and is related to hyperglycemia, hypertension, hyperlipidemia, pregnancy, nephropathy, and anemia [5–7].

Diabetes causes damage to all the major cells of the retina, vascular cells (endothelial cells and pericytes), and pigment epithelial cells [8]. The vascular disruptions in DR are characterized by abnormal autoregulation of retinal blood flow caused by the loss of the pericytes that normally regulate vessel calibre, breakdown of the inner blood-retinal barrier, thickening of the capillary basement membrane, and damage and proliferation of endothelial cells. Characteristic clinical manifestations are the result of four main processes: the appearance of microaneurysms, increased vascular permeability, capillary occlusion, and fibrous and neovascular proliferation. Fluid leakage can range from microexudates to the most severe form, namely, macular edema, which can seriously reduce vision. The leakage of blood cells and platelets through capillary walls cause, intraretinal haemorrhaging. Another lesion characteristic of DR is capillary occlusion (nonperfusion with retinal ischemia), which may lead to the proliferation of new vessels (neovascularization), seeking out new routes to irrigate the ischemic area. These new vessels are often surrounded by fibrous tissue, and this fibrovascular complex may adhere to the posterior part of the vitreous body. Traction on the vitreous which usually happens with age or with rapid eye movement during sleep can rupture the fragile structure of the new vessels and lead to vitreous haemorrhaging or even retinal detachment. New vessels and fibrous tissue can also close the anterior chamber angle which leads to neovascular glaucoma with severe elevations in intraocular pressure (IOP) [8, 9]. 

The primary goal of DR treatment is to improve or protect vision by reducing vascular leaking and macular edema formation, retinal ischemia, and growth of fragile new vessels and thereby preventing vitreous hemorrhages and tractional retinal detachment. However, it should be kept in mind that DR can progress towards advanced stages asymptomatically before actually affecting visual acuity [3, 8, 9].

The retina is a metabolically active tissue, and therefore hyperglycemia in diabetes with associated relative or absolute insulin deficiency is thought to adversely affect its normal physiology. Various biochemical, hemorheological, and immunological mechanisms have been implicated to explain the vascular disruption in retinopathy [10–13]. Recently, numerous clinical and laboratory investigations have identified inflammation as an important factor in the development of DR [14–17]. 

## 2. Inflammation and Diabetic Retinopathy

There is increasing evidence that inflammatory processes have a considerable role in the pathogenesis of DR with multiple studies showing an association of various systemic as well as local (vitreous and aqueous fluid) inflammatory factors and the progression of DR. Inflammation is present in the development of both major causes of impaired vision in diabetes, namely, increased retinal vascular permeability (diabetic macular edema (DME)) and neovascularisation (proliferative diabetic retinopathy (PDR)) [17–19].

Extensive research has verified the potential role of inflammatory mediators in DR. One of these mediators is tumor necrosis factor-alpha (TNF-*α*), a proinflammatory cytokine which is known as an initiator of inflammatory reactions. High TNF-*α* levels have been detected in vitreous, serum, and ocular fibrovascular membranes in patients with DR [20–22]. TNF-*α* gene polymorphism is associated with increased susceptibility to the disease [23]. Similarly, interleukin-1 beta (IL-1*β*) and its downstream signalling molecule caspase 1 are found in high concentrations in the vitreous and retina of diabetic patients [21, 24, 25]. Interleukin-6 (IL-6) levels in the vitreous are significantly correlated with the severity and progress of DR [26, 27]. In patients with PDR, increased vitreous concentrations of the IL-1*β*, IL-6, soluble IL-2 receptor (sIL-2R), and IL-8 were found [28], whilst the serum of the same patients contained elevated levels of TNF-*α*, IL-6, IL-8, and sIL-2R [12, 28, 29]. The mean serum TNF-*α*, IL-8, and sIL-2R levels increased with the stage of diabetic retinopathy with the highest levels being detected in patients with the proliferative form [12]. Recently, some studies have pointed out the role of proinflammatory cytokine IL-12 as a result of local retinal production in the development of DR [30, 31]. Chemokines such as CCL2, CCL5, CXCL8, CXCL10, and CXCL12 are also upregulated in the vitreous samples of patients with DR [32–34]. In the serum of DR patients, levels of TNF-*α*, IL-1*β*, CCL5, and CXCL12 are also found to be significantly increased [21, 34], suggesting that systematic inflammatory reactions are in some respect related to this disease. 

In addition to increases in the above-mentioned inflammatory mediators, increases in adhesion molecules such as intracellular adhesion molecule-1 (ICAM-1) and vascular cell adhesion molecule-1 (VCAM-1) as well as activation of leukocytes have been found to be related to the progression of DR [34, 35]. Both molecules ICAM-1 and VCAM-1 promote chemoattraction of leukocytes into the vascular walls and their migration into retinal tissues [35, 36]. Activated leukocytes via adhesion molecules attach to the vascular epithelium causing the release of inflammatory cytokines, growth cytokines, and vascular permeability factors and thus altering endothelial junctional proteins and allowing leukocytic diapedesis into the retina [14, 17]. Besides disrupting the inner retinal barrier and compromising the blood-retinal barrier (BRB), leukocytes promote angiogenesis. In all these activities, leukocytes further additionally contribute by producing VEGF, an inflammatory mediator that increases vascular permeability and induces the expression of ICAM-1 and VCAM-1, respectively [35, 36]. 

## 3. Treatment of Diabetic Retinopathy

The first step in managing DR is to reduce the risk of its development and progression by controlling the underlying risk factors, namely, hyperglycemia, hypertension, and hyperlipidemia (Table 1). The Diabetes Control and Complications Trial (DCCT) and United Kingdom Prospective Diabetes Study (UKPDS) have shown that intensive glycemic control is associated with a lower risk of retinopathy compared to conventional therapy in type 1 and type 2 diabetes [37, 38]. The UKPDS also determined that intensive blood pressure control reduces microvascular complications compared with less intensive control [39]. Lipid-lowering therapy particularly reducing the levels of serum cholesterol, low-density lipoprotein (LDL), and triglyceride decrease the severity of retinal hard exudates as well as the risk of developing PDR [40, 41]. In addition to controlling these modifiable risk factors, regular dilated eye examinations have been shown to reduce the incidence of blindness due to diabetic retinopathy through early detection and timely treatment [42]. However, irrespective of all these efforts not all cases of DR can be prevented which thereby further highlights the need for effective treatment strategies. 

Currently, laser photocoagulation is the primary method of treatment for patients with diabetic retinopathy who are at a high risk for vision loss but unfortunately is not always effective for improving vision. In many cases, when retinal damage and vision loss have already occurred, laser treatment can simply maintain vision and avoid further vision loss. It is established that in about 50% of patients retinopathy progresses despite laser photocoagulation. The procedure is uncomfortable, and often repeated treatments are required. Moreover, laser photocoagulation is an ablative destroying retinal tissue procedure, where scars always enlarge over time leading to decrease in night vision, colour vision, and peripheral vision as well as loss of 1 or 2 lines of visual acuity in some patients [43, 44]. Pars plana vitrectomy (PPV) is a microsurgical procedure designed to remove vitreous gel usually in order to achieve access to a diseased retina. It is the main method of treating severe complications of PDR such as severe persistent vitreous hemorrhages and tractional retinal detachment [45].

## 4. Pharmacological Treatment of DR

Despite standard intervention, vision loss due to DR still occurs at a frightening rate. Considering the limitations and side effects of current treatments of DR, there has been a continuing attempt to understand the molecular mechanisms that contribute to the occurrence and changes seen in the diabetic retinas. Thus, many researchers have directed their efforts towards better understanding of the microvascular changes in DR in order to develop more effective pharmacologic prevention and treatment as well as establishing new treatment strategies. Currently, the three major classes of pharmacologic agents being studied are corticosteroids, vascular endothelial growth factor (VEGF) antagonists, and agents that are involved in biochemical pathways (polyol pathways activation, diacylglycerol, protein kinase C (PKC) pathway activation, stimulation of cellular oxidative stress, and changes in macromolecule structure and function via the formation of advanced glycation end-products (AGE)) [46, 47].

Considering the involvement of the inflammatory processes of low-grade chronic inflammation in the pathogenesis of DR, special attention is focused on pharmacological agents with anti-inflammatory effect. Inhibiting the inflammatory pathway could be an appealing treatment option for DR in future practices [16–19, 48].

## 5. Inflammatory Mediators and Medical Treatment 

Since inflammation is identified as a relevant mechanism for DR development, significant effort has been directed to the development of new concepts for the prevention and treatment of DR acting on the inflammatory processes (Table 2). 

### 5.1. Corticosteroids

Corticosteroids have been included in the treatment of DR and DME due to their anti-inflammatory and antiangiogenic effects. Various inflammatory mediators that are upregulated in DR and play a significant role in its pathogenesis including TNF-*α*, IL-1*β*, and VEGF are very well modulated with corticosteroids. They have been shown to reduce vascular permeability, reduce the breakdown of the blood retinal barrier, inhibit leukocyte adhesion to vascular walls, and inhibit VEGF gene transcription and translation. They rapidly decrease macular edema; however, their short term and transitory effectiveness limit their application. Frequently, new injections are necessary at different time intervals when the antiedematous effects cease depending on the half-life of the steroid being used. Systemic corticosteroids are excluded from DR therapy due to ocular complications such as cataract formation, IOP, and glaucoma as well as systematic side effects including exacerbation of diabetes. These side effects also occur with intraocular formulations and may additionally limit their application. Currently, several different steroids are being used to treat DME, namely, triamcinolone, fluocinolone, and dexamethasone [49–51].

Intravitreal injection of *triamcinolone acetonide* (IVTA) (Kenalog 40), a slow-releasing steroid, is a promising therapy treatment for DME that suppresses inflammation, reduces vascular leakage, and inhibits fibrovascular proliferation [52]. In eyes with DME, it causes a reduction in foveal thickness and shows improvement in visual acuity in several case series [53, 54]. Intravitreal administration of TA is also an effective treatment options for PDR [55]. However, the treatment effect only lasts approximately 6 months; therefore, repeated treatments may often be required with a risk limit on the safety due to multiple entries in the vitreous. Possible complications of using TA include elevation of IOP, cataract, and endophthalmitis [55]. The most significant complication of IVTA is elevation of IOP resulting in secondary open-angle glaucoma, which sometimes may be severe and intractable [56]. Elevation of IOP up to 24 mm Hg may occur in about 40% of patients, usually within 3 months [57]. Furthermore, cataract formation may become visually significant in about 50% of eyes within 1 year [58]. The rates of injection-related endophthalmitis following IVTA have been reported to range between 0.09% and 0.87% per injection [59]. Retinal detachment, lens trauma, and vitreous hemorrhage are rarely reported complications of IVTA or other intravitreal injections. The use of peribulbar, rather than intravitreal, TA offers reduced risks of endophthalmitis and perhaps other complications; however, this form of administration has some limitations and is less effective than IVTA [60]. 

The lack of efficacy for chronic use associated side effects and the need for reinjections have led to the development of novel sustained release intravitreal steroid delivery methods. These slow release formulations avoid the need for repeated injections, enabling the use of smaller amounts of corticosteroids with less secondary side effects [46, 47, 49, 50, 61]. 

A steroid drug delivery system in development for use in DME is the TA implant (I-vation), which has already completed a Phase I trial in the long-term treatment of DME, and a Phase II clinical trial is being planned. I-vation is composed of biodegradable polymers which slowly degrade over time, thereby bypassing the risk of secondary surgical complications upon removal as compared to nonbiodegradable devices. Other corticosteroids which have been incorporated into these devices include dexamethasone and fluocinolone [61, 62]. 

Ozurdex is an extended-release biodegradable dexamethasone intravitreal implant that has been recently approved by the Food and Drug Administration (FDA) for the treatment of macular edema secondary to retinal vein occlusions (RVO) and noninfectious uveitis. A Phase III clinical trial of Ozurdex use in the treatment of DME is currently underway. A previous study found that 700 *μ*g dexamethasone was well tolerated and produced statistically significant improvements in BCVA and central retinal thickness at Day 90; however, at Day 180 no significant difference in visual acuity was found, and both treatments groups (350 *μ*g and 700 *μ*g) had an increased incidence of elevated IOP [62].

The fluocinolone acetonide intravitreal implant (Retisert) is FAD-approved for the treatment of chronic, noninfectious posterior segment uveitis. A Phase III clinical trial conducted in patients with DME reported large cases of cataract and glaucoma. [62, 63]. Another potential new steroid delivery system is the sustained release fluocinolone acetonide nonbiodegradable intravitreal insert (Iluvien) which is designed to release the drug gradually up to three years. Since the device is miniature, it can be injected into the back of the eye with a 25-gauge needle creating a self-sealing hole with the procedure being similar to an intravitreal injection [62, 64]. The two ongoing pivotal multicenter trials known collectively as FAME study have shown improvement in best corrected visual acuity (BCVA) at low-dose insert (0.19 mg total, approx. 0.23 *μ*g/day) out of the two doses being studied [62, 65]. 

### 5.2. Vascular Endothelial Growth Factor (VEGF) Inhibitors

Vascular endothelial growth factor (VEGF), primarily isoform VEGF_164/165_, is a potent vasoactive cytokine and a key mediator of blood-retina barrier breakdown and angiogenesis in the ischemic retina. VEGF is produced by the pigment epithelial cells, citespery, and endothelial cells of the retina in response to hypoxia from capillary loss and/or microaneurysm formation [4, 8, 66, 67]. 

The VEGF levels are significantly elevated in patients with DME when compared with nondiabetic eye conditions and its intravitreal concentration increases with the progression of DR from nonproliferative form to active PDR [66, 67]. Similarly, successful panretinal photocoagulation (PRP) reduced intraocular VEGF levels by 75% in patients treated for ocular neovascularization [67]. These data suggest that specific inhibition of VEGF activity may prevent retinal neovascularization and associated blood flow abnormalities. The role of VEGF in retinal neovascularization has encouraged the development of drugs that directly inhibit the VEGF molecule such as the anti-VEGF aptamer, pegaptanib (Macugen), the monoclonal antibody fragment Ranibizumab (Lucentis), and the full-length antibody bevacizumab (Avastin) [46, 47]. 

#### 5.2.1. Pegaptanib

Pegaptanib (Macugen) is a selective aptamer directed against the VEGF-A 165 isoform. It was the first United States FDA-approved ophthalmologic anti-VEGF agent for the treatment of choroidal neovascularization in age-related macular degeneration (AMD) [68]. In phases 1, 2, and 3 prospective clinical trial, pegaptanib appeared to improve anatomic and visual outcomes in patients with DME as well as regressing neovascularisation [69, 70]. 

#### 5.2.2. Bevacizumab

Bevacizumab (Avastin), a full-length recombinant humanized antibody, is active against all isoforms of VEGF-A. It is FDA-approved as a systemic treatment for metastatic colorectal cancer [71]. Case reports and small, nonrandomized pilot studies have documented efficacy of using off-label intravitreal bevacizumab against exudative AMD, macular edema from nonischemic central retinal vein occlusion, iris neovascularization as well as diffuse DME, and various complications of PDR [72, 73]. The Diabetic Retinopathy Clinical Research network (DRCR.net) in 2007 completed phase 2 of a prospective randomized multicenter clinical trial to determine the safety and possible benefits of this agent [74]. 

#### 5.2.3. Ranibizumab

Ranibizumab (Lucentis), a recombinant humanized antibody fragment, is active against all isoforms of VEGF-A. Intravitreal ranibizumab is FDA-approved for the treatment of exudative AMD in 2006 [75]. Two pilot studies of ranibizumab demonstrated efficacy in the treatment of DME [76, 77]. The results of phase 3 of two prospective randomized multicenter comparing clinical trials, one in patients with DME without PDR and another in patients with DME and PDR (RISE and RIDE), have been recently published. The outcome of phase 3 confirmed the results of phase 2 with the conclusion that ranibizumab rapidly and sustainably improved the vision, reduced the risk of further vision loss, and improved macular edema in patients with DME with low rates of ocular and nonocular impairments [78]. 

Notwithstanding all efforts, the results of multiple conducted researches have not yielded promising results for the use of anti-VEGF treatments in DME such as in PDR or neovascular glaucoma. This clinical observation therefore indicates that the pathogenesis of DME is multifactorial, and many other factors beyond VEGF may play a role in this process.

### 5.3. Miscellaneous Anti-Inflammatory Agents 

#### 5.3.1. Nonsteroidal Anti-Inflammatory Drug (NSAID)

NSAID are a group of drugs including aspirin and salicylate that exert anti-inflammatory activity by inhibiting cyclooxygenase (COX) enzyme-mediated eicosanoid formation [79]. The Early Treatment DR Study and the Dipyridamole Aspirin Microangiopathy of Diabetes Study [80] showed that although treatment of patients with advanced DR with a low dose of aspirin does not have any benefits, the development of retinal microaneurysms is significantly minimised in patients with early stage of DR when treated with a high dose of aspirin (900 mg/day). Clinical trial of specific COX-2 inhibitors has been discouraged given that systemic COX-2 inhibitors increase incidence of heart attacks and strokes [79]. However, in preclinical studies topical administration of COX-2 inhibitor was shown to reduce signs of DR similar to its systematic application without the side effects [81], and therefore it would be worthy to investigate its therapeutic benefits in future clinical trials.

#### 5.3.2. Effect on Oxidative Stress

As a key mediator in inflammation, oxidative stress serves as an important target for anti-inflammatory therapy. In diabetic rats, supplements of antioxidants such as vitamins C and E attenuate the development of acellular capillaries and decrease the number of pericyte ghosts [82]. In diabetic animals or models of ischemic retinopathy, blocking increased activity of nicotinamide adenine dinucleotide phosphate (NADPH) oxidase induced by diabetes prevents vascular leakage and pathological neovascularization, respectively. Moreover, these studies also reveal that statin treatment effectively blocks upregulation of NADPH oxidase activity in DR, suggesting that the benefit of statin treatment is at least partly due to its activity in blocking NADPH oxidase [83].

#### 5.3.3. Blocking RAS (Renin-Angiotensin System)

The RAS could be a promising aim for DR treatment since this system is involved in both diabetes and hypertension-induced retinal inflammation and is a pathway that interrelates with multiple other pathways including oxidative stress and AGEs. Specific blockade of the RAS with AT1R blocker (Losartan, Candesartan) and angiotensin-converting enzyme inhibitor (Enalapril) has been shown to prevent oxidative stress, inflammation, and vascular damage in diabetic animal models [14, 84] as well as reducing the risk and the progression of retinopathy [14, 85, 86]. However, further trials are necessary to resolve and to fully evaluate the beneficial effect of RAS blockade before its clinical usefulness is fully understood.

#### 5.3.4. Blocking Inflammatory Molecules

In addition to blockers of general inflammation, studies have been performed to determine whether targeting specific inflammatory molecules can be beneficial in DR treatment. As a key player in many inflammatory reactions, the proinflammatory cytokine TNF-*α* may be a suitable target for many inflammatory diseases.

Etanercept (Enbrel) is a recombinant fusion protein having anti-TNF-*α* property and is FDA-approved for the treatment of psoriasis [87]. A small series of patients with refractory DME were treated with intravitreal etanercept, but no statistically significant improvement was recorded [88]. Infliximab (Remicade) is another TNF-*α* antagonist that is FDA-approved to treat Crohn's disease. An investigation of systemic treatment of DME with Infliximab has led to a study of administration through intravitreal injection [89]. 

## 6. Conclusion

Diabetic retinopathy is a major cause of blindness in developed countries and remains one of the most serious complications of diabetes. Thus, recently, many researchers have directed their efforts towards better understanding of the pathogenesis of DR in order to develop new and more effective preventative and treatment strategies with the emphasis being on pharmacologic therapy. However, at this time, primary prevention with intensive glycemic control, strict blood pressure regulation, and lipid-modifying therapy as well as local ocular treatment (laser photocoagulation and pars plana vitrectomy) still remain the proved treatment options addressing diabetic retinopathy. As prospective randomized clinical trials accumulate data, the role and guidelines of pharmacologic treatments will become clearer. The discovery that inflammation plays a critical role in the pathogenesis of DR opens up new pathways and methods for the development of novel improved treatments and strategies for this devastating disease. Neither the precise mechanism by which the inflammatory cascade is initiated and involved in DR occurrence and development nor the individual roles of particular inflammatory molecules in the different stages of DR are currently fully understood. Some inflammatory molecules have multiple functions and different actions on the various pathways of inflammatory processes which open up the challenge of finding an effective drug target. Alternatively, drugs targeting several mechanisms simultaneously may be more efficient, and therefore more studies are necessary to understand the endogenous anti-inflammation processes in DR.

## Figures and Tables

**Table 1:** Treatment of diabetic retinopathy.

Controlling the risk factors	Local ocular treatment	Pharmacologic treatment
Hyperglycemia	Laser photocoagulation of retina	Corticosteroids
Hypertension	Pars plana vitrectomia	Anti-VEGF treatment
Hyperlipidemia		Agents involved in biochemical pathways

VEGF: vascular endothelial growth factor.

**Table 2:** Anti-inflammatory therapy of diabetic retinopathy.

Drug	Target	Anti-inflammatory mechanism
*Corticosteroids* Triamcinolone acetonide Fluocinolone acetonide Dexamethasone	Glucocorticoide receptor	Proinflammatory transcription factors blockade

*VEGF inhibitors* Pegaptanib Bevacizumab Ranibizumab	VEGF	Blocking VEGF-mediated inflammation, vascular permeability, and angiogenesis

NSAID	COX	Inhibition of proinflammatory, prostaglandins production

Vitamins C, E	Oxidative stress	Antioxidative stress

*Blocking RAS* Losartan, Candesartan, and Enalapril	RAS	Blocking RAS-mediated inflammation

*Blocking inflammatory molecules* Etanercept, Infliximab	TNF-*α*	Blocking TNF-*α*-induced inflammation

VEGF: vascular endothelial growth factor; NSAID: nonsteroidal anti-inflammatory drug; COX: cyclooxygenase; RAS: renin-angiotensin system; TNF-*α*: tumour necrosis factor-alpha.
